# Intracranial control after Cyberknife radiosurgery to the resection bed for large brain metastases

**DOI:** 10.1186/s13014-015-0523-4

**Published:** 2015-10-31

**Authors:** Jennifer Vogel, Eric Ojerholm, Andrew Hollander, Cynthia Briola, Rob Mooij, Michael Bieda, James Kolker, Suneel Nagda, Geoffrey Geiger, Jay Dorsey, Robert Lustig, Donald M. O’Rourke, Steven Brem, John Lee, Michelle Alonso-Basanta

**Affiliations:** Department of Radiation Oncology, University of Pennsylvania, TRC-2 West, 3400 Civic Center Boulevard, Philadelphia, PA 19104 USA; Department of Radiation Oncology, Pennsylvania Hospital, Widener Ground Level, 800 Spruce Street, Philadelphia, PA 19107 USA; Department of Neurosurgery, University of Pennsylvania, 3 Silverstein, 3400 Spruce Street, Philadelphia, PA 19104 USA

**Keywords:** Brain metastases, Radiosurgery, CyberKnife

## Abstract

**Background:**

Stereotactic radiosurgery (SRS) is an alternative to post-operative whole brain radiation therapy (WBRT) following resection of brain metastases. At our institution, CyberKnife (CK) is considered for local treatment of large cavities ≥2 cm. In this study, we aimed to evaluate patterns of failure and characterize patients best suited to treatment with this approach.

**Methods:**

We retrospectively reviewed 30 patients treated with CK to 33 resection cavities ≥2 cm between 2011 and 2014. Patterns of intracranial failure were analyzed in 26 patients with post-treatment imaging. Survival was estimated by the Kaplan-Meier method and prognostic factors examined with log-rank test and Cox proportional hazards model.

**Results:**

The most frequent histologies were lung (43 %) and breast (20 %). Median treatment volume was 25.1 cm^3^ (range 4.7–90.9 cm^3^) and median maximal postoperative cavity diameter was 3.8 cm (range 2.8–6.7). The most common treatment was 30 Gy in 5 fractions prescribed to the 75 % isodose line. Median follow up for the entire cohort was 9.5 months (range 1.0–34.3). Local failure developed in 7 treated cavities (24 %). Neither cavity volume nor CK treatment volume was associated with local failure. Distant brain failure occurred in 20 cases (62 %) at a median of 4.2 months. There were increased rates of distant failure in patients who initially presented with synchronous metastases (*p* = 0.02). Leptomeningeal carcinomatosis (LMC) developed in 9 cases, (34 %). Salvage WBRT was performed in 5 cases (17 %) at a median of 5.2 months from CK. Median overall survival was 10.1 months from treatment.

**Conclusions:**

This study suggests that adjuvant CK is a reasonable strategy to achieve local control in large resection cavities. Patients with synchronous metastases at the time of CK may be at higher risk for distant brain failure. The majority of cases were spared or delayed WBRT with the use of local CK therapy.

## Background

Approximately 20–40 % of patients with cancer will develop intracranial metastases [1]. Treatment options for these patients include surgery, whole brain radiation therapy (WBRT) and stereotactic radiosurgery (SRS). For patients with large intracranial metastases, local control with single modality treatment is poor. Studies have shown <10 % complete response rate with WBRT for tumors approximately 2 cm in diameter [2]. Local control is maintained in less than 50 % of tumors larger than 2 cm after radiosurgery alone [3]. Randomized studies have demonstrated that WBRT following surgical resection of a solitary metastasis decreases the rate of local recurrence within the surgical bed as well as distant recurrence within the brain [4]. However, WBRT is associated with decreased cognitive function and quality of life without a proven overall survival benefit [5, 6]. SRS to the surgical bed is an emerging alternative that may allow WBRT to be deferred for a select group of patients.

Large resection cavities are less amenable to single fraction SRS given the high risk of radiation damage with increasing cavity size [7]. Fractionated SRS following surgical resection has been shown to be feasible and safe utilizing linear accelerator-based and CyberKnife (CK) technologies for cavities greater than 3 cm [8–10]. However, there is limited information on optimal patient selection for this treatment approach. In particular, questions remain regarding risk of local and distant failure, especially leptomeningeal carcinomatosis (LMC). In this study, we report outcomes following single or multi-fraction SRS for large brain metastases after surgical resection. We quantify local control and assess patient and treatment characteristics associated with intracranial failure.

## Methods

### Patient selection

Patients were considered for SRS following surgical resection. Patients recommended for SRS had low intracranial metastatic burden (1–4 metastases) and good overall prognosis as judged by performance status, status of extracranial disease, age, and histology [11]. CK was considered for lesions ≥ 2 cm, lesions with gross residual or recurrent disease, and lesions near critical structures. SRS was ideally performed after full post-operative wound healing 4–6 weeks from the time of resection.

With the approval of the institutional review board, we retrospectively analyzed medical records of all patients treated with single or multiple fraction CyberKnife (CK) SRS to surgical beds of intracranial metastases ≥2 cm at the time of radiosurgery. Cases were performed between 2011 and 2014 at the Pennsylvania Hospital (University of Pennsylvania Health System). Patients with a history of WBRT prior to CK or with a plan for CK followed by WBRT were excluded from this study. Patients with synchronous unresected lesions treated with SRS alone were included in this study.

### Data collection

All clinical data was obtained from electronic medical records and Social Security Death Index. The status of extracranial disease was defined as none, stable, or progressive based on systemic imaging prior to CK. Those who were diagnosed at the time of intracranial disease or who had no treatment to their primary site were characterized as newly diagnosed. Post-operative MRI was obtained 24–48 h after resection in each case. Gross total resection (GTR) was defined as no residual enhancing tumor after surgery on MRI. Graded prognostic assessment (GPA) class was assigned to each patient according to methods described by Sperduto et al. [11].

### Radiosurgical technique and follow up

The CyberKnife (Accuray, Sunnyvale, CA) was used to deliver all radiosurgical treatments. Brain MRI was obtained at 1-mm slices with gadolinium contrast. The MRI scan was fused to the computed tomography scan (CT) for target delineation. The gross tumor volume (GTV) was contoured as the edge of the resection cavity including contrast enhancement. The clinical tumor volume (CTV) was an expansion of 2–3 mm margin added to the GTV. Planning target volume (PTV) was the same as the CTV.

Dose was based on the size of the PTV and preference of the treating physician. Larger cavities were typically treated to 30 Gy in five fractions and smaller cavities to 24 Gy in three fractions as has been previously described [12]. With a plan maximal dose of 100 %, doses were prescribed to the 55–90 % isodose lines. The PTV was covered by at least 95 % of the dose in all cases. Conformality index was typically between 1.1 and 1.2, and smaller than 1.3. The number of beams was between 100 and 200 and treatment times were kept under 1 h.

After CK, follow-up included clinical visits at 1 month and then 3-month intervals. Surveillance MRI was obtained 2–3 months following CK and subsequently every 3 months. Local failure was defined as new and progressive nodular enhancement in the resection bed over a minimum follow-up of 4 months, or two consecutive MRI scans. Radionecrosis was evaluated using dynamic contrast-enhanced perfusion imaging (DCE-MRI) and magnetic resonance spectroscopy (MRS). Radionecrosis rather than local progression was suggested by 1) low cerebral blood volume 2) increased lactate/creatine ratio and decreased choline/creatine ratio and 3) regression or stability over a minimum follow-up of 4 months without additional treatment [13, 14]. Clinical decisions regarding local progression versus radionecrosis were made in a multidisciplinary setting including input from a diagnostic radiologist. Distant failure was defined as new brain metastases away from the surgical cavity or development of LMC, diagnosed based on MRI, clinical symptoms, and/ or cerebrospinal fluid analysis.

### Statistical analysis

Statistical tests were performed using Stata 13.1 (StataCorp). Overall survival was estimated using the Kaplan-Meier method with all cases included in the analysis. Living patients or those lost to follow-up were censored at the date of last clinical encounter. Local and distant failure was examined by the Kaplan-Meier method in cases where post-CK imaging was available. Patients who received salvage WBRT were censored at last MRI prior to WBRT for the failure analysis. Univariable analyses were computed using log-rank test and a *p* value ≤ 0.1 was considered statistically significant. Multivariable analysis was performed via the Cox proportional hazards ratio and *p* ≤ 0.05 was considered statistically significant. For this exploratory, hypothesis-generating study we made no adjustment for multiple comparisons.

## Results and discussion

A total of 30 patients were treated with CK to 33 resection cavities. Fifteen patients (50 %) were male and 15 (50 %) were female. The most frequent histologies were lung (43 %) and breast (20 %). Median GPA was 2.5 (range 1–4). Gross total resection (GTR) was achieved in 19 (58 %) cases. A median of one synchronous metastasis was observed in 10 patients (range 1–3) [Table 1].

**Table 1:** Patient characteristics

Characteristic	Value
Sex^a^	
Male (%)	15 (50)
Female (%)	15 (50)
Age in years at CK	
Median (range)	58 (28–97)
Primary tumor^a^	
NSCLC (%)	12 (40)
SCLC (%)	1 (3)
Breast (%)	6 (20)
Melanoma (%)	4 (13)
Other (%)	7 (23)
Extracranial disease^a^	
Newly diagnosed/ progressing (%)	15 (50)
None/ stable (%)	15 (50)
Median GPA class^a^	
Breast	2 (1.5–3.5)
NSCLC/SCLC	2.5 (1.5–3.5)
Melanoma	3 (2–3)
GI	2 (2–4)
Other	2.75 (1–3.5)
Postoperative diameter in cm	
Median (range)	3.8 (2.8–6.7)
Surgery extent^b^	
GTR (%)	19 (58)
STR (%)	14 (42)
Synchronous intact mets^a^	
Yes (%)	10 (33)
No (%)	20 (67)
Weeks from resection to CK	
Median (range)	7 (4–19)

*Preop* preoperative, *CK* Cyberknife, *GTR* gross total resection, *STR* subtotal resection, *mets* metastases, *NSCLC* non-small cell lung cancer, *SCLC* small cell lung cancer, *GI* gastrointestinal

^a^ *N* = 30 patients

^b^ *N* = 33 cases

SRS was performed a median of 7 weeks from resection (range 4–19 weeks). Causes of delay included wound infection (*n* = 1), pulmonary embolism (*n* = 1), treatment of primary site (*n* = 1), systemic staging (*n* = 2), insurance referral delay (*n* = 2), and intracranial re-staging (*n* = 7). At the time of treatment planning, interval growth was observed in 13 cavities.

The median SRS dose to the resection cavity was 30 Gy (range 16–35) delivered in 1 to 5 fractions (median, 5 fractions). Median cavity volume was 17.5 cm^3^ (range 2.4–69.8 cm^3^) and median treatment volume was 25.1 cm^3^ (range 4.7–90.9 cm^3^). Median maximal postoperative cavity diameter was 3.8 cm (range 2.8–6.7) [Table 2].

**Table 2:** Fractionation schemes

No. of fractions	N	Median dose (Gy)	Median dose per fraction (Gy)	Median diameter (cm)	Median isodose line (%)
1	2	18.5 (16–21)	18.5 (16–21)	2.8 (2.8–2.9)	61 (55–66)
3	5	24 (24)	8 (8)	3.6 (3.1–4.5)	76 (71–79)
5	26	30 (25–35)	6 (5–7)	4.2 (3.2–6.7)	76 (64–88)

*No.* number, *PTV* planning treatment volume

The median clinical follow up for the entire cohort of 30 cases was 9.5 months (range 1.0–34.3), and for the 13 living patients was 16.9 months (range 4.4–35.2). Twenty-six patients with 29 treated cavities were followed with serial imaging.

**Fig. 1:**
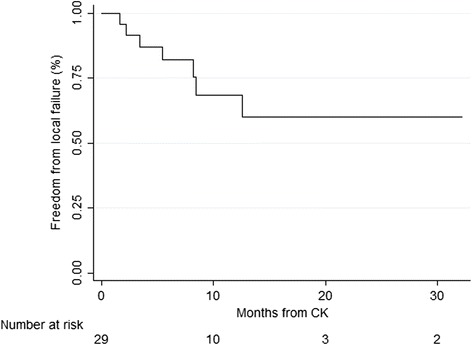
Freedom from local intracranial progression after CK

Local failure occurred in 7 cavities, for a crude rate of 24 %. Median time to local failure was 3.4 months in the cases that failed. Six-month and one-year actuarial local control were 82.3 and 68.5 % [Fig. 1]. Six of the 7 cases with local failure had images available for evaluation. Failure occurred within the GTV in 5 instances and 3 mm from the GTV in 1 case. Evaluation of preoperative tumor diameter, postoperative tumor diameter, GTV, and PTV at a variety of cut-off points showed no association with local failure (*p* > 0.2).

**Fig. 2:**
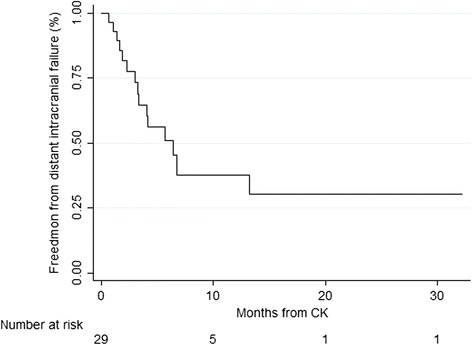
Freedom from distant intracranial progression after CK

Of the 26 patients with available follow-up imaging, distant brain failure occurred in 16 (62 %) at a median of 4.2 months from CK. Actuarial 6-month and 1-year distant brain failure were 55.3 and 73.9 % [Fig. 2]. On univariable analysis, presence of synchronous metastases at the time of diagnosis (*p* = 0.04), dose per fraction <6 Gy (*p* = 0.07), and total dose <30 Gy (*p* = 0.1) were associated with distant failure. On multivariable analysis, synchronous metastases at diagnosis was significantly associated with distant failure (*p* = 0.02, 95 % CI 1.33–14.5).

Among the 26 cases with follow-up imaging, 9 developed leptominengeal disease, for a crude rate of 34 %. On univariable analysis, simultaneous resection of multiple metastases (*p* = 0.01) and >50 days delay to CK (*p* = 0.03) were associated with LMC. There was no association with tumor histology, cavity diameter, cavity volume, or cavity location (*p* > 0.3). On multivariable analysis, no variable was significantly associated with LMC.

**Fig. 3:**
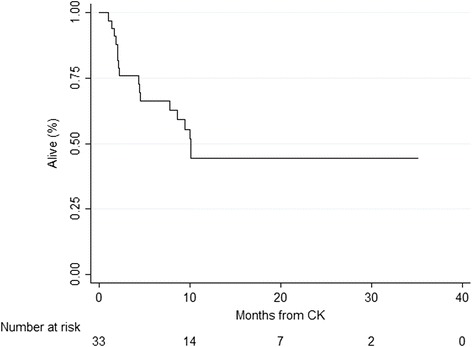
Survival after CK

By Kaplan-Meier analysis, the median overall survival was 10.1 months from CK [Fig. 3]. On univariable analysis, male gender (*p* = 0.01), the presence of synchronous metastases (*p* = 0.07), GPA ≤ 3 (*p* = 0.04) and age >60 years (*p* = 0.1) were statistically significantly associated with overall survival. On multivariable analysis, male gender remained statistically significant (*p* = 0.01).

**Fig. 4:**
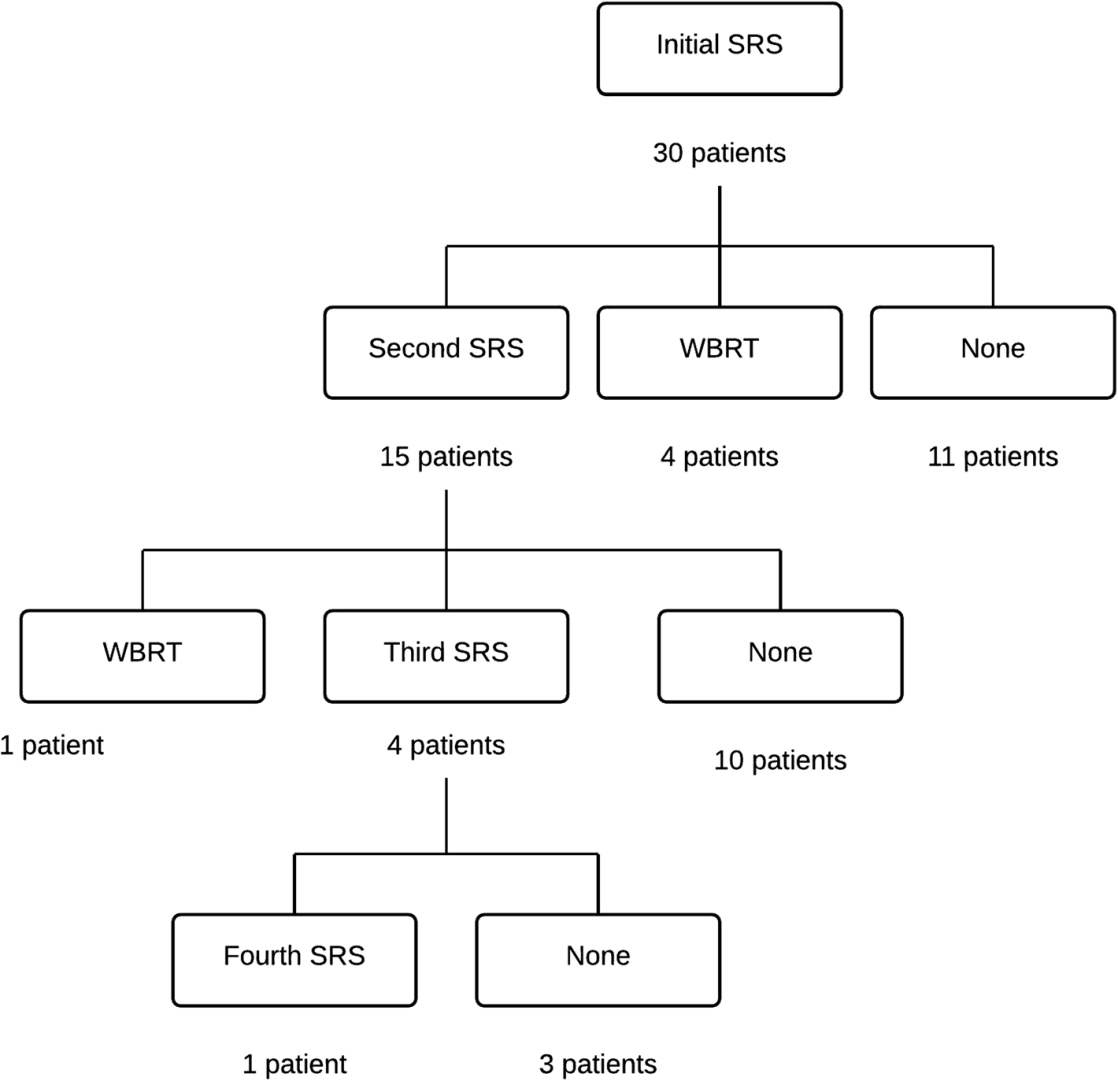
Outcomes after Initial SRS

Two patients underwent upfront resection of two simultaneous lesions. Eight were treated with concurrent SRS for synchronous, unresected metastases found at the time of CK. Seven patients underwent additional SRS [Fig. 4]. Five of 30 total patients (17 %) were treated with salvage WBRT at a median of 5.2 months from CK. Patients with leptomeningeal disease were significantly more likely to receive salvage WBRT (*p* = 0.001).

Three patients developed clinically significant radionecrosis requiring treatment with steroids (10 %). None had neurosurgical resection. Two patients had new onset seizures within 3 months of CK.

## Discussion

For patients treated with surgical resection of intracranial metastases, whole brain radiation therapy has been shown to improve intracranial control without an overall survival benefit [5, 7]. Neurocognitive decline and decreased quality of life have been associated with whole brain radiation and led to increasing use of local therapies to delay or defer WBRT after resection [6].

Numerous studies have reported outcomes of single fraction stereotactic radiosurgery to small resection cavities in patients without prior WBRT [Table 3]. One year actuarial local control rates range from 78 to 100 % and distant failures occur in 33–66 % of patients [15, 16, 17, 18]. Whole brain radiation is reported as salvage therapy in approximately one third of patients.

**Table 3:** Literature of single fraction SRS to the resection cavity

Authors & year	No. cavities	Treatment modality	Median dose (Gy)	Crude LF (%)	DF (%)	Salvage WBRT (%)	LMC (%)	MS (mos)
Iwai et al., 2008 [19]	21	GK	17	24	48	NR	24	20
Do et al., 2009 [26]	33	LINAC	15	12	63	47	NR	12
Jagannathan et al., 2009 [27]	47	GK	19	7	87	28	NR	11
Karlovits et al., 2009 [28]	52	LINAC	15	8	55	31	NR	15
Limbrick et al., 2009 [29]	16	GK	20	27	60	40	NR	20
Hwang et al., 2010 [30]	25	GK	15–20^a^	0	33	NR	NR	15
Kalani et al., 2010 [31]	68	GK	15	21	NR	NR	NR	13.2
Jensen et al., 2011 [15]	112	GK	17	13	54	37	7.5	10.9
Rwigema et al., 2011 [20]	77	CK	18	26	47	26	NR	14.5
Kelly et al., 2012 [32]	18	LINAC	18	11	35	24	NR	N/A
Ogiwara et al., 2012 [33]	56	GK	16	9	38	14	NR	21
Prabhu et al., 2012 [22]^b^	64	LINAC	18	17	NR	NR	NR	13
Robbins et al., 2012 [16]	85	LINAC	16	19	55	35	8.2	12
Broemme et al., 2013 [23]^b^	44	LINAC	17^c^, 24^d^	9	66	36	NR	15.9
Hartford et al., 2013 [35]	49	LINAC	10	16	63	45	NR	NR
Luther et al., 2013 [17]	120	GK	16	14	40	NR	NR	NR
Brennan et al., 2014 [34]	40	LINAC	18	30	47	NR	NR	14.7
Ojerholm et al., 2014 [18]	96	GK	16	18	54	33	14	22.3

*No.* number, *GK* Gamma Knife, *LINAC* linear accelerator, *CK* Cyberknife, *LF* local failure, *DF* distant failure, *MS* median survival, *LMC* leptomeningeal carcinomatosis, *mos* months, *NR* not reported, *N/A* not applicable

^a^Reported range

^b^Majority single fraction SRS

^c^Median single fraction dose

^d^Median multi fraction dose

Large lesions are less amenable to single fraction treatment given increasing dose to surrounding normal tissue and risk of radiation necrosis, especially for lesions >3 cm [5]. Previous studies have established safety and efficacy of fractionated SRS in treatment of large brain metastases, with rates of radionecrosis from 2 to 6 % [8–10]. However, there are few reports describing outcomes for patients with large resection cavities treated with fractionated SRS without WBRT [Table 4]. Reported cases are characterized by cavity volumes ranging from 8.7 to 29.5 cm^3^ and low rates of radionecrosis, from 3 to 9 % [19–23]. Series of patients treated with predominantly fractionated regimens report 71–91 % local control, comparable to single fraction series. Similarly, approximately one third of patients are salvaged with whole brain radiation.

**Table 4:** Literature of single or multiple fraction SRS to resection cavity

Authors & year	No. cavities	Treatment modality	Median dose (Gy)	Crude LF (%)	DF (%)	Salvage WBRT (%)	LMC (%)	MS (mo)
Choi et al., 2012 [21]^a^	102	CK	16^b^	11	53	28	NR	15.6
Minniti et al., 2013 [24]	101	LINAC	27	8	53	27	NR	17
Ling et al., 2015 [25]	100	CK	22	NR	64.1	33	6	12.7
Present study	33	CK	30	24	62	17	34	10.1

*No.* number, *CK* Cyberknife, *LINAC* linear accelerator, *LF* local failure, *DF* distant failure, *MS* median survival, *LMC* leptomeningeal carcinomatosis, *mos* months, *NR* not reported

^a^Majority multi fraction SRS

^b^Biologically equivalent single fraction dose

In some series, increased risk of local failure is seen with increasing preoperative diameter or cavity size [Table 5]. Brennan et al. report 39.1 % local failure in patients with tumors ≥3 cm as compared to 7.5 % with maximal diameter <3 cm [24]. Prabhu et al. report a hazard ratio of 1.04 for local recurrence with increasing PTV volume [25]. In these series, patients were predominantly treated with single fraction SRS. Our series consisted of patients with large lesions treated most often with fractionated SRS. Increased risk of local failure was not seen in association with increasing tumor diameter, increasing cavity size, or larger treatment volumes.

**Table 5:** Significantly associated clinical characteristics

Authors	LF	DF	LMC	OS
Iwai et al. [19]	Dose < 18Gy	N/A	Infratentorial location	N/A
Jagannathan et al. [27]	Greater treated volume	N/A	N/A	Systemic progression
Karlovits et al. [31]	N/A	N/A	N/A	>1 intracranial metastasis
				Extracranial disease
Jensen et al. [15]	Preop diameter > 3 cm	Dose <35Gy	N/A	N/A
Rwigema et al. [20]	Greater PTV	N/A	N/A	Age
				RPA score
Choi et al. [21]	N/A	Melanoma histology	N/A	KPS Preop diameter
Ogiwara et al. [33]	N/A	N/A	N/A	Extracranial metastases
Prabhu et al. [22]	Larger PTV volume	N/A	N/A	N/A
	Marginal dose <18 Gy			
Brennan et al. [34]	NSCLC histology	Infratentorial location	N/A	N/A
	Preop diameter > 3 cm			
	Dural/ pial involvement			
Hartford et al. [35]	Preop diameter ≥ 2 cm	Preop diameter ≥ 2 cm	N/A	GPA ≤1
Ojerholm et al. [18]	Preop diameter ≥ 3 cm	N/A	Breast histology	New diagnosis or untreated primary
			Infratentorial location	
Ling et al. [25]	N/A	Uncontrolled systemic disease	N/A	Increasing no. metastases
		Melanoma histology		Uncontrolled systemic disease
		Increasing no. brain metastases		
Present study	N/A	Synchronous metastases	N/A	Male gender

*LF* local failure, *N/A* not applicable, *PTV* planning treatment volume, *Preop* preoperative, *LMC* leptomeningeal carcinomatosis, *CK* Cyberknife, *OS* overall survival, *RPA* recursive partitioning analysis, *KPS* Karnofsky performance status, *GPA* graded prognostic assessment, *no.* number

Distant failures in reported series of fractionated stereotactic radiosurgery range from 53 to 64 % [26, 27, 28]. In our series, distant failure occurred in 62 % of treated patients. Our study did not show any correlation between cavity size and increased distant failure, although we did observe increased rates of distant failure with synchronous metastases at the time of resection. Other studies have shown a similar trend towards increased distant failure when multiple metastases are present at the time of initial SRS [29, 30]. As in other series, the majority of patients were salvaged with additional SRS and spared WBRT.

Rates of distant failure from leptomeningeal carcinomatosis have been less frequently reported. Given the lack of data for this entity, our study adds to the literature in regards to rates after local therapy. For patients treated with single fractions, rates range from 4.5 to 24 % [31, 32, 33, 34]. A single report of patients treated predominantly with multi-fraction SRS demonstrated a rate of 6 % [35]. Our rate of 34 % is higher than that reported for surgical cavities treated with CK and GK. This risk has been associated previously with breast histology and infratentorial location [18, 19]. In this cohort, histology, location, and cavity size had no association with risk of LMC. Clinical factors associated with LMC remain to be characterized.

In our series, we observed an association between male gender and overall survival which was independent of age, RPA classification, presence of synchronous metastases, and tumor histology. This association has not been previously observed in series of SRS to resection cavities. However, male gender has been reported as an independent prognostic factor in analyses by Serizawa et al. for patients treated with GK to multiple simultaneous brain metastases [36, 37].

This study is limited by small patient numbers and its retrospective nature. Patient selection was biased towards those with higher performance status and lower metastatic burden as compared to those who may have been offered WBRT alone. Long-term neurocognitive function and death due to neurologic progression were not available for comparison to regimens using WBRT. Doses delivered were done at the discretion of the treating physician, although the study is strengthened by institutionally based planning constraints and follow-up.

Reports of single and multi-fraction SRS using CK remain largely from retrospective single institution series. Ongoing studies comparing postoperative SRS to observation (MD Anderson Cancer Center 2009–0381) and WBRT (Intergroup N107C) will further clarify the role of SRS after resection of a single metastasis. Neoadjuvant SRS is an alternate treatment algorithm for local control in large brain metastases. Phase I/II studies are evaluating the role of neoadjuvant therapy for resectable lesions (NCT01891318) and future studies may incorporate SRS into treatments for brain metastases which are unresectable at presentation. Findings from these studies will continue to clarify which patients are most suitable candidates for local therapy following resection of a brain metastasis and to evaluate alternate treatment paradigms in this setting.

## Conclusions

Adjuvant SRS to the resection cavity for patients with large brain metastases provides local control while sparing patients potential long-term neurocognitive risk of WBRT. Local and distant control can be achieved independent of tumor diameter and cavity volume. Patients with synchronous metastases may have higher rates of distant failure when treated with local therapies alone.

## Abbreviations

WBRT: Whole brain radiation therapy
SRS: Stereotactic radiosurgery
LMC: Leptomeningeal carcinomatosis
CK: CyberKnife
RPA: Recursive partitioning analysis
MRI: Magnetic resonance imaging
GTR: Gross total resection
CT: Computed tomography
GTV: Gross tumor volume
CTV: Clinical target volume
PTV: Planning target volume
Preop: Preoperative
STR: Subtotal resection
mets: Metastases
NSCLC: Non-small cell lung cancer
SCLC: Small cell lung cancer
GI: Gastrointestinal
No.: Number
LF: Local failure
DF: Distant failure
MS: Median survival
mos: Months
NR: Not reported
N/A: Not applicable
